# Gambogic acid protects LPS-induced apoptosis and inflammation in a cell model of neonatal pneumonia through the regulation of TrkA/Akt signaling pathway

**DOI:** 10.1186/s40360-021-00496-9

**Published:** 2021-05-11

**Authors:** Xu Gao, Jingya Dai, Guifang Li, Xinya Dai

**Affiliations:** 1 Department of Pharmacy, Cangzhou People’s Hospital, Cangzhou, Hebei Province 061000 China; 2 Department of Pharmacy, Cangzhou Central Hospital, Cangzhou, Hebei Province 061001 China

**Keywords:** Neonatal pneumonia, gambogic acid, lipopolysaccharide, inflammation, apoptosis

## Abstract

**Objective:**

In this work, we investigated the effects of gambogic acid (GA) on lipopolysaccharide (LPS)-induced apoptosis and inflammation in a cell model of neonatal pneumonia.

**Method:**

Human WI-38 cells were maintained *in vitro* and incubated with various concentrations of GA to examine WI-38 survival. GA-preincubated WI-38 cells were then treated with LPS to investigate the protective effects of GA on LPS-induced death, apoptosis and inflammation. Western blot assay was utilized to analyze the effect of GA on tropomyosin receptor kinase A (TrkA) signaling pathway in LPS-treated WI-38 cells. In addition, human AKT serine/threonine kinase 1 (Akt) gene was knocked down in WI-38 cells to further investigate the associated genetic mechanisms of GA in protecting LPS-induced inflammation and apoptosis.

**Results:**

Pre-incubating WI-38 cells with low and medium concentrations GA protected LPS-induced cell death, apoptosis and inflammatory protein productions of IL-6 and MCP-1. Using western blot assay, it was demonstrated that GA promoted TrkA phosphorylation and Akt activation in LPS-treated WI-38 cells. Knocking down Akt gene in WI-38 cells showed that GA-associated protections against LPS-induced apoptosis and inflammation were significantly reduced.

**Conclusions:**

GA protected LPS-induced apoptosis and inflammation, possibly through the activations of TrkA and Akt signaling pathway. This work may broaden our understanding on the molecular mechanisms of human neonatal pneumonia.

**Supplementary Information:**

The online version contains supplementary material available at 10.1186/s40360-021-00496-9.

## Introduction

Neonatal pneumonia is a serious respiratory infectious disease often occurring in neonate patients with high risk of morbidity and mortality [1, 2]. The incidence rate of neonatal pneumonia may vary, approximately from 1 to 35 %, depending on various factors, such as race, gestational age, level of received neonatal care or socioeconomic environment [1, 3]. The characteristic diagnosis of neonatal pneumonia includes bacteria-induced respiratory infection and may be discovered as wet lung rales using a stethoscope [2, 4]. The typical treatment plans include antiviral and antibacterial approaches, such as with erythromycin 12.5 mg/kg orally every 6 h for 14 days or azithromycin 20 mg/kg orally/IV once a day for 3 days (https://www.msdmanuals.com/professional/pediatrics/infections-in-neonates/neonatal-pneumonia). However, in developing countries such as China, antibiotic abuse has significantly increased the strain resistance among neonatal pneumonia patients who received conventional therapies [1, 5–8]. Thus, it is critical to understand the full molecular mechanisms underlying neonatal pneumonia, in order to develop novel and efficient treatment plans.

It has been well established that natural compounds may have significant anti- antibacterial functions. Among them, gambogic acid (GA) is a xanthonoid isolated from the exudate of Garcinia hanburyi Hook f. (Clusiaceae) and has been commonly used in clinical settings as multi-module reagents [9]. For example, GA had been demonstrated to suppress cancer development by inhibiting ubiquitin-proteasome signal pathway [10]. In addition, GA has fracture-healing function by inducing osteoblastic differentiation [11]. Moreover, GA has been demonstrated to protect neuronal death through neurotrophic signaling pathway as well as activating tropomyosin receptor kinase A (TrkA) receptor [12]. Specifically, a recent study showed that GA had markedly anti-inflammatory and anti-apoptosis activities in lipopolysaccharide (LPS)-induced mastitis [13]. However, any possible molecular mechanisms of GA in human neonatal pneumonia have never been elucidated.

In the present study, we established an *in vitro* cellular model of human neonatal pneumonia, in which a human lung fibroblast cell line, WI-38 cells were cultured *in vitro* and treated with LPS to induce neonatal pneumonia-like inflammation and apoptosis among WI‐38 cells [13–18]. We then pre-incubated WI‐38 cells with various concentration of GA to observe the potential anti-inflammatory and anti-apoptosis effects of GA on LPS-induced cytotoxicity. In addition, we adapted genetic engineering approach to knock down human AKT serine/threonine kinase 1 (Akt) gene in WI-38 cells, to further explore the molecular and genetic mechanisms of GA in protecting LPS-induced inflammation and apoptosis in this cellular model of neonatal pneumonia.

## Materials and methods

### Cell model of neonatal pneumonia with treatment of GA

An *in vitro* cell culture model of neonatal pneumonia was established according to previous studies [15, 18, 19]. Briefly, a diploid human lung fibroblast cell line, WI-38 cells, was commercially acquired from the American Type Culture Collection (ATCC, USA), and maintained in 6-well plate containing Minimum Essential Medium (Thermo Fisher Scientific, USA) supplemented with 10 % fetal bovine serum (FBS, Sijiqing Biotechnology, Hangzhou, China) and Penicillin-Streptomycin (1000 U/mL, Thermo Fisher Scientific, USA) in a tissue-culturing environment at 37 °C with 5 % CO_2_. Lipopolysaccharide (LPS) was commercially acquired (MilliporeSigma, Shanghai, China) and applied at the concentration of 10 mg/mL to the WI‐38 cells for 24 h. Gambogic acid (GA) was also commercially acquired (MilliporeSigma, Shanghai, China) and applied to the WI‐38 cells at concentrations of 1, 5, 10, 20, 50, 100, 200 and 500 nM for 24 h prior to LPS treatment.

### Viability assay

WI-38 cells were lifted from culture, suspended, centrifuged and stained with 0.4 % Trypan Blue (Thermo Fisher Scientific, USA) for 30 min 37 °C. Then, cell viability, or the percentage of live cells, was measured using a Countess™ Automated Cell Counter (Thermo Fisher Scientific, USA) according to the manufacturer’s instruction.

### Apoptosis assay

WI-38 cell apoptosis was characterized using a Click-iT Plus TUNEL Alexa Fluor 594 dye Assay (Thermo Fisher Scientific, USA) according to the manufacturer’s instruction. Also, a 4’-6-diamidino-2-phenyindole (DAPI) antibody (Thermo Fisher Scientific, USA) was applied to identify WI‐38 cell nuclei. The percentage of apoptotic (TUNEL-positive) WI‐38 cells were measured among all (DAPI-positive) WI‐38 cell populations.

### Western blot assay

Total protein was extracted from WI-38 cells using the Pierce IP Lysis buffer (Thermo Fisher Scientific, USA) according to the manufacturer’s instruction. Equal amount of proteins from each sample were then subjected to separation using the SDS-PAGE gel (Thermo Fisher Scientific, USA) and then transferred using the PVDF membrane (Thermo Fisher Scientific, USA). After sealing with 5 % BSA, the membranes were incubated with primary antibodies for 24 h at 4°C. The primary antibodies are, mouse monoclonal to Interleukin 6 (IL-6, 1:1,000, Invitrogen, USA), mouse monoclonal to monocyte chemotactic protein-1(MCP-1, 1:2,000, Invitrogen, USA), mouse monoclonal to tropomyosin receptor kinase A (TrkA, 1:1,000, Invitrogen, USA), rabbit polyclonal to TrkA (phospho Y490) (p-TrkA, 1:200, Invitrogen, USA), mouse monoclonal to AKT serine/threonine kinase 1 (Akt, 1:1,000, Invitrogen, USA) and rabbit polyclonal to β-actin (1:5,000, Invitrogen, USA). Subsequently, the membranes were cultured with suitable horseradish peroxidase-conjugated goat anti mouse (or rabbit) secondary antibody (10,000, Abcam, USA) for 4 hour at 4°C. For visualization and quantification of the bands, an IBright CL1500 (Thermo Fisher Scientific, USA) was used according to the manufacturer’ instruction.

### Akt knockdown assay

A siRNA specifically targeting human Akt gene (si_Akt), and a non-specific control siRNA (si_C) were commercially obtained from RiboBio (RiboBio Technology, Guangzhou, China). WI-38 cells were transfected with si_Akt or si_C using Lipofectamine 3000 (Thermo Fisher Scientific, USA) for 24 h, followed by qRT-PCR to verify the gene knockdown efficiency.

### RNA extraction and quantitative real-time PCR (qRT-PCR)

Total RNAs was extracted from WI-38 cells using a TRIzol™ Plus RNA Purification Kit (Invitrogen, USA) according to the manufacturer’s instruction. Reverse Transcription was conducted using a High-Capacity cDNA Reverse Transcription Kit (Invitrogen, USA) according to the manufacturer’s instruction. For quantitative real-time PCR (qRT-PCR) on human Akt gene, a PowerUp™ SYBR™ Green Master Mix (Invitrogen, USA) was used on a QuantStudio qRT-PCR system (Applied Biosystems, USA). Relative gene expression levels were characterized using the 2^(−ΔΔCt)^ method.

### Statistical analysis

All data were collected using biologically independent triplicates or more repeats. The averaged values were presented as means +/- S.E.M. For statistical analysis, a Windows-based SPSS software (SPSS, version 18.0, USA) was used, and data were compared using one-way ANOVA. A difference measurement with *P* value < 0.05 was determined as significant.

## Results

### GA protected LPS-induced WI-38 cell death

**Fig. 1:**
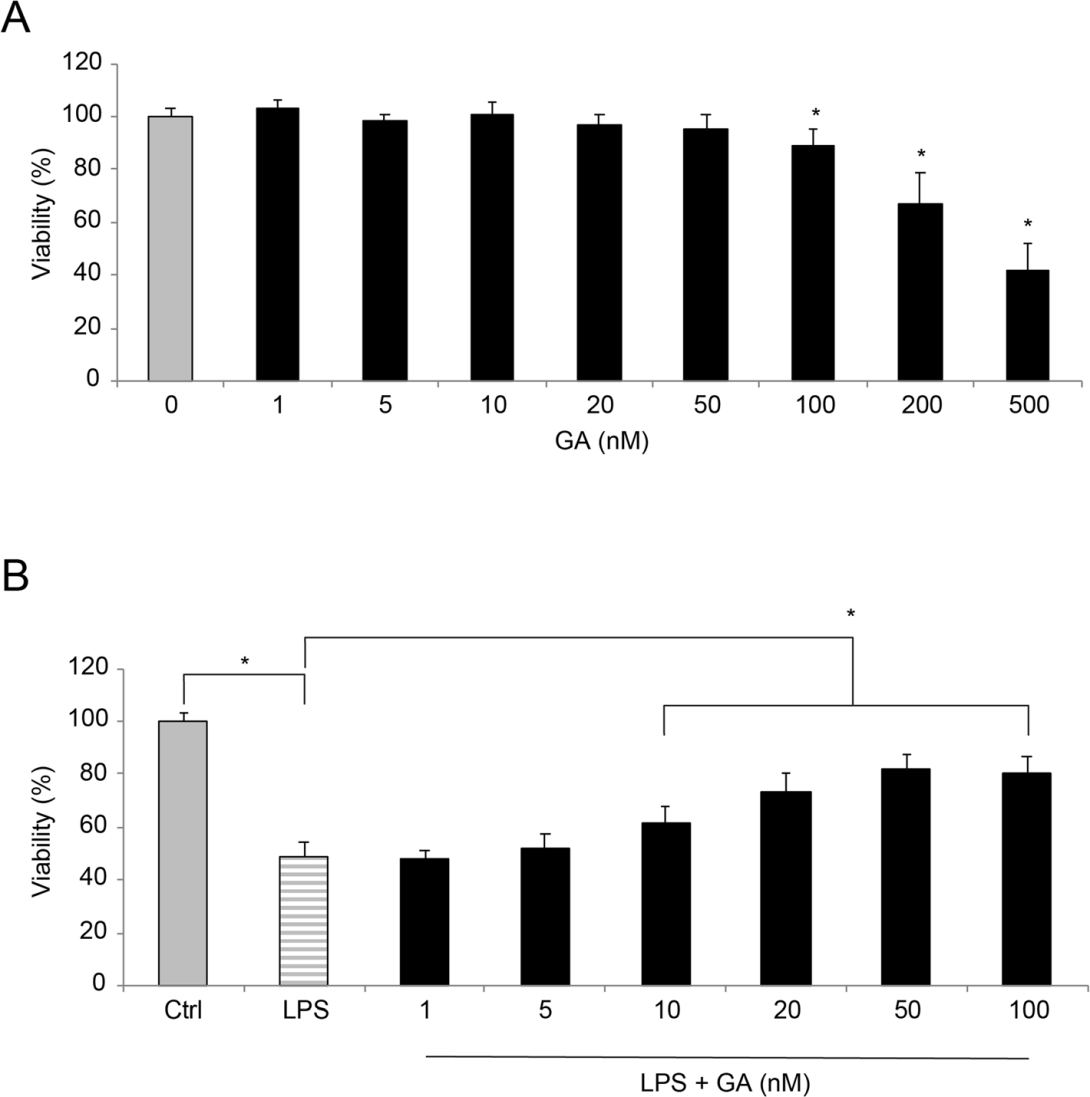
The effect of GA on LPS-induced WI-38 cell death. aWI-38 cells were cultured in vitro, and incubated with Gambogic acid (GA) at concentrations of 0, 1, 2, 5, 10, 20, 50, 100, 200 and 500 nM for 24h. Then, potential cell death caused by GA was characterized using a viability assay (* P < 0.05). bAfter GA pre-incubation, WI-38 cells were treated with 10mg/ml lipopolysaccharide (LPS) for 24h. Again, potential cell death was characterized using a viability assay (* P < 0.05)

WI-38 cells were cultured *in vitro* and incubated with Gambogic acid (GA) at various concentrations for 24 h. A viability assay demonstrated that, while at high concentrations ( > = 100 nM), GA induced cell death among WI-38 cells (Fig. 1**a**, * *P* < 0.05).

Then, after WI-38 cells were incubated with GA for 24 h, they were treated with 10 mg/ml lipopolysaccharide (LPS) for 24 h. Using a viability assay, we noticed that LPS alone (with 0 nM GA pre-incubation) induced significant WI-38 cell death, whereas 10 ~ 100 nM GA pre-incubation markedly protected LPS-induced WI-38 cell death (Fig. 1**b**, * *P* < 0.05). Thus, for the rest of the study, GA was used at the concentration of 50 nM, as it did not induce WI-38 cell death but had significant protection against LPS-induced cell death.

### GA rescued WI-38 cell from LPS-induced apoptosis and inflammatory injury

**Fig. 2:**
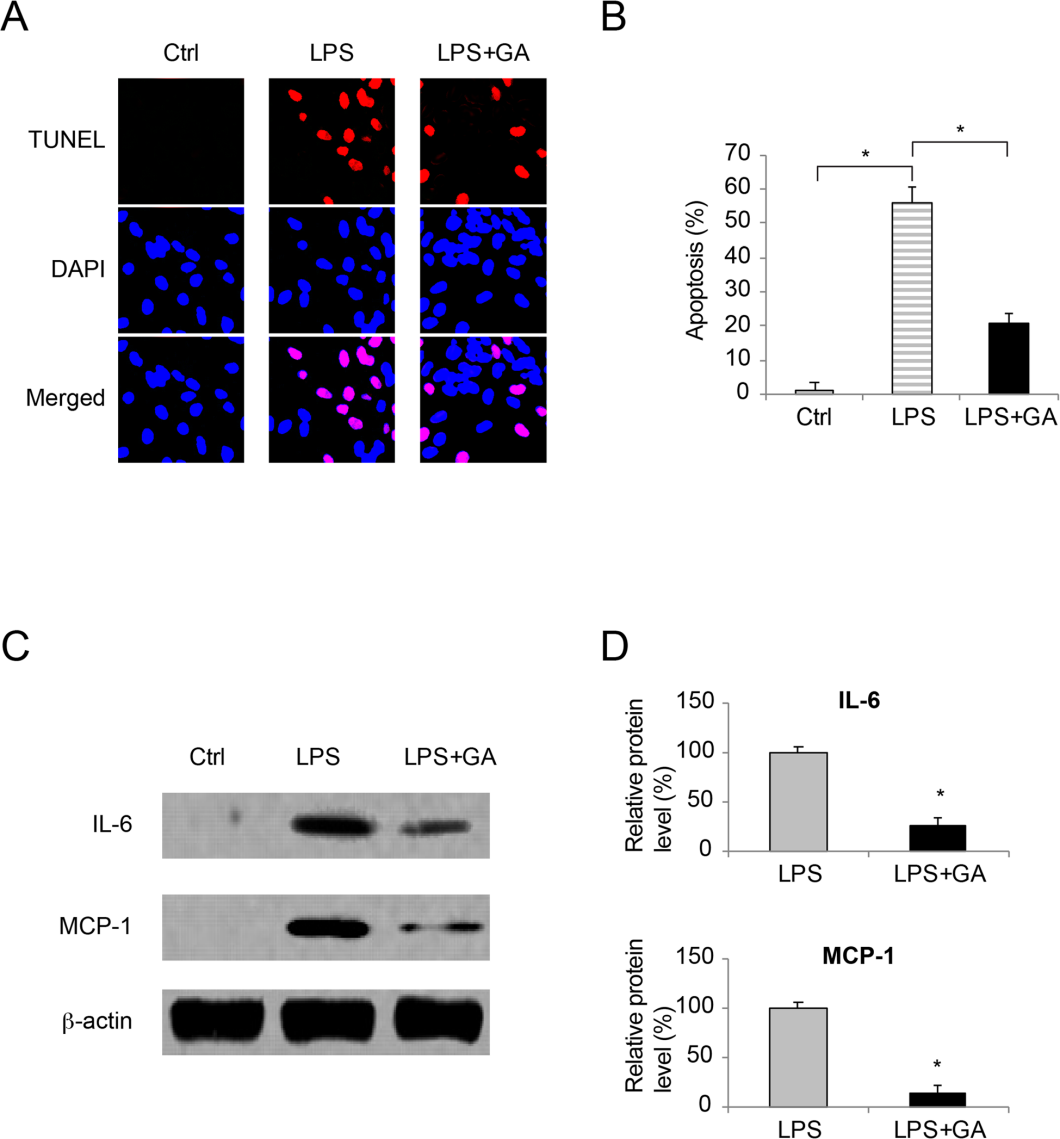
The effect of GA on LPS-induced WI-38 apoptosis and inflammation.aWI-38 cells were cultured in vitro (Ctrl), or treated with 10mg/ml LPS for 24h (LPS), or pre-incubated with 50 nM GA for 24h prior to LPS treatment (LPS + GA). A TUNEL assay was applied to identify apoptotic cells with TUNEL (Red) immunoreaction. During the meantime, DAPI (Blue) immunoreaction was applied to identify WI-38 cell nuclei. bFor images acquired in TUNEL assay, the percentages of apoptotic WI-38 cells were compared among Ctrl, LPS and LPS + GA conditions (* *P* < 0.05). cFor WI-38 cells under Ctrl, LPS and LPS + GA conditions, western blot analysis were conducted to compare IL-6 and MCP-1 protein expressions. dFor western blot data in (c), relative band intensities of IL-6 and MCP-1 were compared between LPS and LPS + GA conditions (* *P* < 0.05)

WI-38 cells were either untreated (Ctrl), treated with 10 mg/ml LPS for 24 h (LPS) or pre-incubated with 50 nM GA for 24 h prior to LPS treatment (LPS + GA). After that, a TUNEL assay demonstrated that, as compared to control condition (Ctrl), LPS induced significant TUNEL immunoreaction, whereas GA pre-incubation reduced LPS-induced TUNEL immunoreaction among WI-38 cells (Fig. 2**a**). Analysis on the percentage of apoptotic WI-38 cells confirmed that, LPS caused significant apoptosis, whereas GA pre-incubation rescued LPS-induced apoptosis in WI-38 cells (Fig. 2**b**, * *P* < 0.05).

In addition, the effect of GA on LPS-induced inflammatory injury was characterized by western blot assay. It showed, under control condition, little or none IL-6 or MCP-1 protein was produced in WI-38 cells (Fig. 2**c,***Ctrl*). Under LPS treatment, significant IL-6 and MCP-1 protein production was spotted in WI-38 cells (Fig. 2**c**, *LPS*). However, while LPS-treated WI-38 cells were pre-incubated with GA, IL-6 or MCP-1 protein productions were significantly reduced (Fig. 2**c and d**, *LPS*, * *P* < 0.05).

### GA activated TrkA/Akt in LPS-injured WI-38 cells

**Fig. 3:**
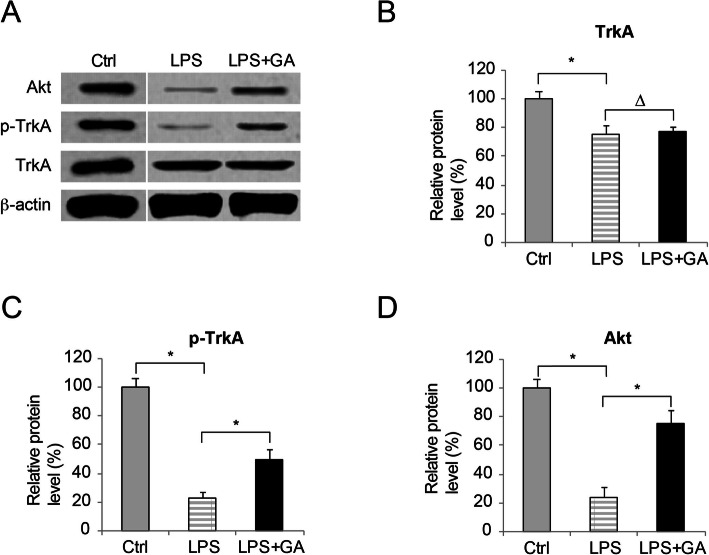
The effect of GA on TrkA/Akt in LPS-treated WI-38 cells. aWI-38 cells were cultured in vitro (Ctrl), or treated with 10mg/ml LPS for 24h (LPS), or pre-incubated with 50 nM GA for 24h prior to LPS treatment (LPS + GA). Western blot analysis was conducted to examine protein productions of TrkA, p-TrkA and Akt. bFor blot result in (A), TrkA protein expressions were quantitatively compared between WI-38 cells under Ctrl, LPS and LPS + GA conditions (∆ P > 0.05). cFor blot result in (A), phosphor-TrkA (p-TrkA) protein expressions were quantitatively compared between WI-38 cells under Ctrl, LPS and LPS + GA conditions (* P < 0.05). dFor blot result in (A), Akt protein expressions were quantitatively compared between WI-38 cells under Ctrl, LPS and LPS + GA conditions (* P < 0.05)

Next, we sought the signaling pathways to be responsible for the protecting effect of GA in LPS-injured WI-38 cells. As GA might act as a selective agonist for TrkA receptor [12], we decided to investigate whether TrkA/Akt signaling pathway was activated by GA in LPS-injured WI-38 cells. Western blot analysis demonstrated that, protein productions of TrkA, p-TrkA and Akt were all reduced by LPS treatment (Fig. 3**a, b, c and d**, *Ctrl vs. LPS*, * *P* < 0.05). Between LPS-treated WI-38 cells and those pre-incubated with GA prior to LPS treatment (LPS + GA), protein productions of TrkA were not different (Fig. 3**a and b**, *LPS vs. LPS + GA*, * *P* < 0.05). However, it showed that GA pre-incubation significantly increased the protein productions of phosphor-TrkA (Fig. 3**a and c**, *LPS vs. LPS + GA*, * *P* < 0.05), and AKT (Fig. 3**a and d**, *LPS vs. LPS + GA*, * *P* < 0.05).

### Akt downregulation reversed the rescuing effect of GA in LPS-injured WI-38 cells

**Fig. 4:**
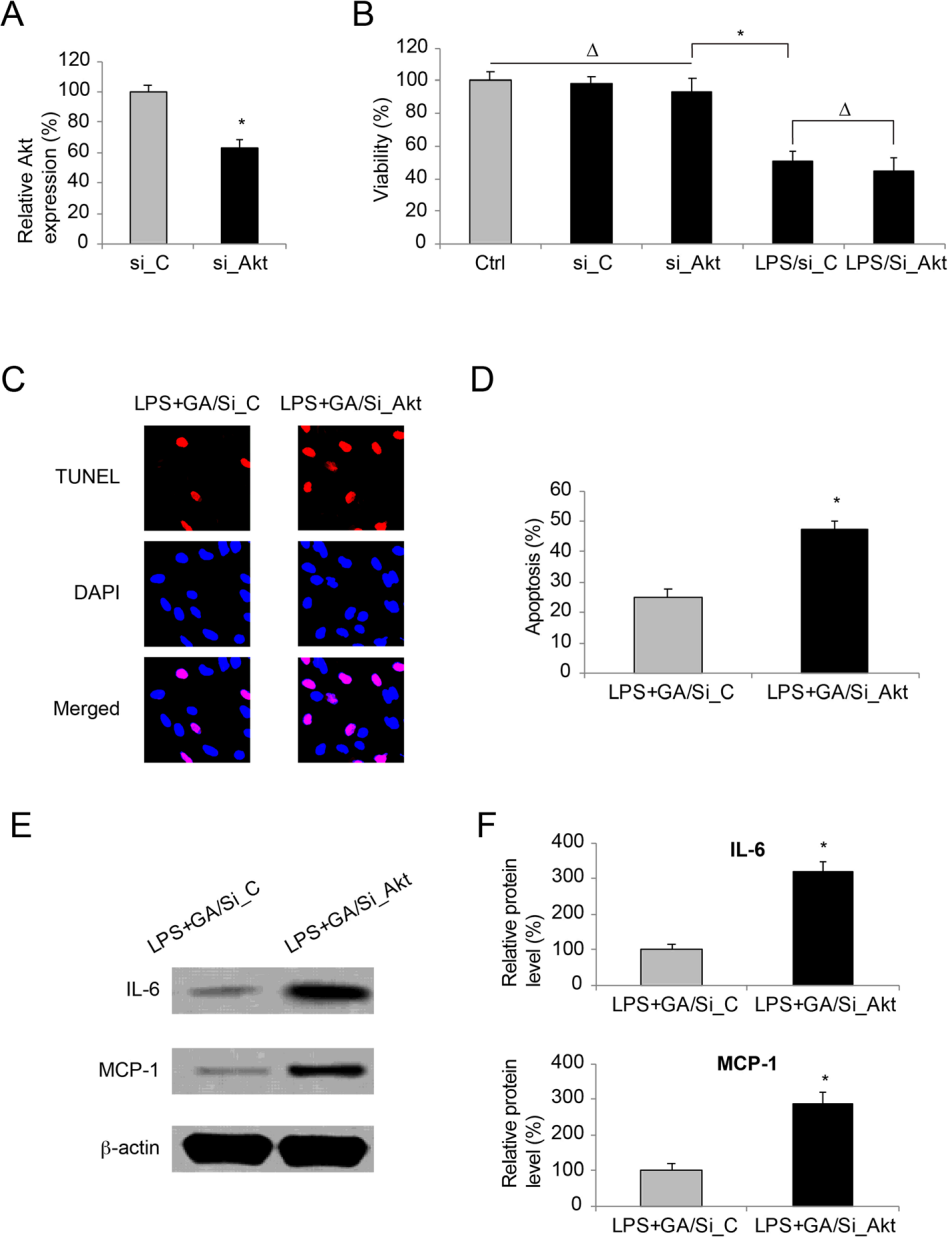
The effect of Akt knockdown in WI-38 cells double-treated with GA and LPS. aWI-38 cells were transfected with a siRNA specifically targeting human Akt gene (si_Akt), and a non-specific control siRNA (si_C). Then, a qRT-PCR assay was performed to compare WI-38 endogenous Akt expressions (* P < 0.05). bCell viabilities was compared among WI-38 cells under control (ctrl) condition (not transfected by siRNA, nor treated with LPS), WI-38 cells transfected with si_C or si_Akt, and siRNA-transfected WI-38 cells treated with 10mg/ml LPS for 24h (* P < 0.05; ∆ P > 0.05). cSiRNA-transfected WI-38 cells were pre-incubated with 50 nM GA for 24h and then treated with 10mg/ml LPS for another 24h (LPS + GA). A TUNEL assay was applied to identify apoptotic cells with TUNEL (Red) immunoreaction. Also, DAPI (Blue) immunoreaction was applied to identify WI-38 cell nuclei. dFor siRNA-transfected WI-38 cells which were treated with LPS + GA, the percentages of apoptotic WI-38 cells were compared (* P < 0.05). eFor siRNA-transfected WI-38 cells which were treated with LPS + GA, western blot analysis was conducted to examine IL-6 and MCP-1 protein expressions. fFor western blot data in (E), quantitative assessment on relative band intensities of IL-6 and MCP-1 were performed (* P < 0.05)

Finally, we sought whether Akt was the key component responsible for the rescuing effect of GA in LPS-injured WI-38 cells. To examine this hypothesis, a siRNA targeting human Akt gene, si_Akt was transfected into the culture of WI-38 cells. The result of qRT-PCR demonstrated that, as compared to WI-38 cells received transfection of a non-specific siRNA (si_C), those received si_Akt transfection had significantly low Akt expression (Fig. 4 **a**, * *P* < 0.05). Then, viability was assessed in siRNA-transfected WI-38 cells. It showed siRNA transfection did not cause cell death in WI-38 cells (Fig. 4**b**, *Ctrl vs. si_C vs. si_Akt*, ∆ *P* > 0.05). Also, siRNA transfection did not alter the toxic effect of LPS on WI-38 cells (Fig. 4**b**, * P < 0.05). In addition, in LPS-treated WI-38 cells, it showed cell viabilities were not different between those transfected with si_C and those transfected with si_Akt (Fig. 4**b**, *LPS/si_C vs. LPS/si_Akt*, ∆ *P* > 0.05).

Then, siRNA_transfected WI-38 cells were pr-incubated with 50 nM GA for 24 h, followed by another 24 h treatment of 10 mg/ml PLS. After that, the TUNEL assay showed that, more TUNEL immunoreaction was detected in si_Akt-transfected WI-38 cells then in si_C-transfected WI-38 cells (Fig. 4 **c**). Analysis on the percentage of apoptotic WI-38 cells confirmed that, Akt downregulation reversed the protective effect of GA pre-incubation on LPS-induced WI-38 apoptosis (Fig. 4**d**, * *P* < 0.05). In addition, western blot assay was conducted on these WI-38 cells. It showed, significantly more IL-6 and MCP-1 protein production was spotted in si_Akt-transfected WI-38 cells, then in si_C-transfected WI-38 cells (Fig. 4**e**). Quantification on western blot densitometry confirmed this finding, demonstrating that Akt downregulation reversed the reducing effect of GA pre-incubation on LPS-induced inflammatory protein productions (IL-6 and MCP-1) in WI-38 cells (Fig. 4 **f**, * *P* < 0.05).

## Discussions

In this work, we discovered that GA had significantly protective effects against LPS-induced cell death, inflammation and apoptosis in WI-38 cells.

First in our work, a viability assay demonstrated that, at low to medium concentrations (< 100 nM), GA did not induce WI-38 cell death. However, at higher concentrations ( > = 100 nM), GA induced significant cell death in the WI-38 culture, in line with previous studies showing high-dose GA lead to cytotoxicity in both animal and human models [20, 21]. Then, we treated GA-preincubated WI-38 cells with LPS under *in vitro* condition and found that LPS-induced cell death was significantly reduced by pre-incubation of 10 ~ 100 nM GA. It was critical for us to carry out the remaining experiments by not confounding any observations with GA’s endogenous toxic effect. For that reason, we used 50 nM GA as the standard concentration for all other experiments in this work.

Second in our work, we investigated the effects of GA on LPS-induced apoptosis and inflammation in WI-38 cells. The results from both TUNEL assay and western blot assay demonstrated that, GA significantly reduced apoptosis and inflammatory protein biomarkers in LPS-injured WI-38 cells. In a very recent study, Tang and colleagues demonstrated that GA could alleviate inflammation and apoptosis and protect the blood-milk barrier in mastitis [13]. However, before our work, the anti-apoptosis and anti-inflammation effects of GA has never been reported in any studies related to neonatal pneumonia.

Further in this work, we sought the downstream signaling pathway associated with the protection of GA in LPS-injured WI-38 cells. Western blot analysis showed that, while TrkA expression were not affected, GA pre-incubation induced TrkA phosphorylation and Akt upregulation. This finding is in line with previous study showing the neurotrophic effect of GA [12]. However, our work is the first-ever report suggesting a therapeutic potential of GA in neonatal pneumonia-related cellular injury. Furthermore, while we specifically knocked down Akt in WI-38 cells, it showed GA-rendered protection against LPS-induced apoptosis and inflammation was significantly ameliorated. This data not only supported the findings in previous publications showing the inflammation-suppressing effect of Akt [22, 23], but also suggested that the anti-cytotoxic mechanism of GA in LPS-treated WI-38 cells was very likely through the Akt signaling pathway. In the recent study in mastitis, it was demonstrated that GA protected the blood-milk barrier and alleviated inflammation / apoptosis by regulating the nuclear factor kappaB (NF-kappaB) signaling pathway [13]. In addition, GA-induced TrkA activation was shown to be correlated with osteoblastic differentiation [11]. Thus, further studies are needed to explore whether other signaling pathways, such as those down-streaming to NF-kappaB, or those associated with bone formation/development, may be interacted with TrkA/Akt signaling pathway to regulate GA-associated protection against cellular injury in neonatal pneumonia.

## Supplementary Information

**Additional file 1.**

**Additional file 2**

## Data Availability

Data are available upon reasonable request.
