# Supplementary figures and images for: Gambogic acid protects LPS-induced apoptosis and inflammation in a cell model of neonatal pneumonia through the regulation of TrkA/Akt signaling pathway
Source: BMC Pharmacol Toxicol. 2021 May 11;22:28. doi: 10.1186/s40360-021-00496-9 (PMC8112032; doi:10.1186/s40360-021-00496-9)

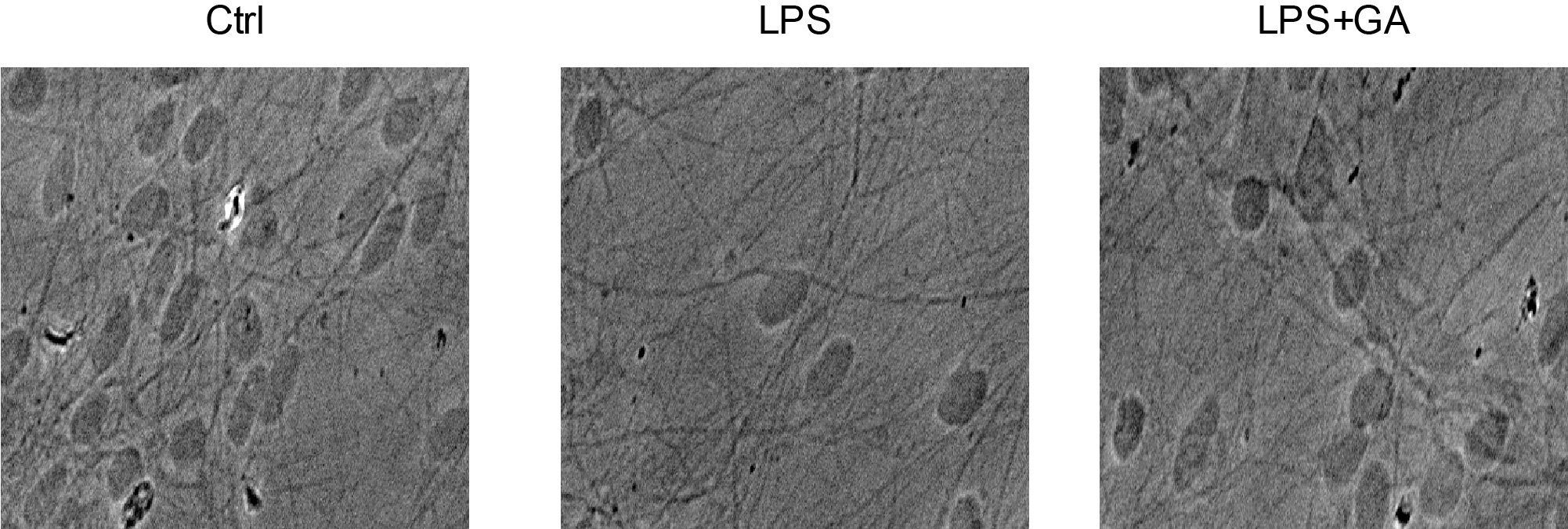

Supplement: Supplementary file 1 — Additional file 1. [file 40360_2021_496_MOESM1_ESM.jpg]

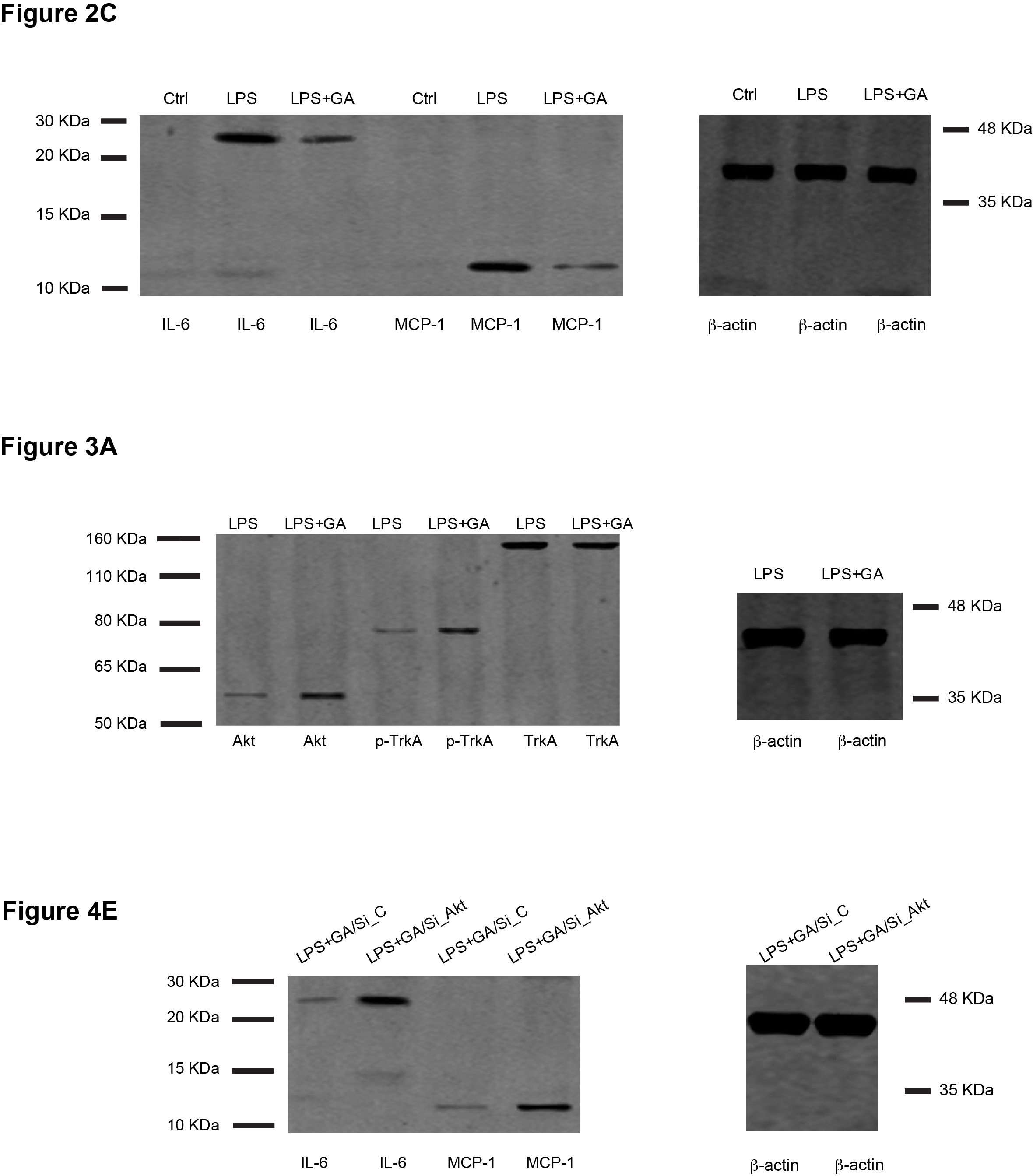

Supplement: Supplementary file 2 — Additional file 2 [file 40360_2021_496_MOESM2_ESM.jpg]
